# Tumor‐infiltrating CD8^+^ T cell is prognostic and predicts adjuvant chemotherapy benefit in patients with limited‐stage small cell esophageal carcinoma

**DOI:** 10.1002/ctm2.456

**Published:** 2021-06-27

**Authors:** Zhihui Zhang, Chaoqi Zhang, Guochao Zhang, Yuejun Luo, Liyan Xue, Qingpeng Zeng, Peng Wu, Lide Wang, Nan Sun, Jie He

**Affiliations:** ^1^ Department of Thoracic Surgery National Cancer Center/National Clinical Research Center for Cancer/Cancer Hospital Chinese Academy of Medical Sciences and Peking Union Medical College Beijing China; ^2^ Department of Pathology National Cancer Center/ National Clinical Research Center for Cancer/Cancer Hospital Chinese Academy of Medical Sciences and Peking Union Medical College Beijing China

To the Editor,

The current study conducted the first exploration of biomarkers for prediction of adjuvant chemotherapy (aCT) benefit in patients with small cell esophageal carcinoma (SCEC). SCEC is a rather rare and aggressive malignant neuroendocrine gastrointestinal tumor. Unlike other two major pathological subtypes of esophageal carcinoma (EC)—esophageal squamous cell carcinoma (ESCC) and esophageal adenocarcinoma (EAC)—SCEC only accounts for ∼1.5% of the different subtypes of ECs.^1^ Southeast Asian countries have the highest incidence rates of SCEC worldwide, especially in China.^2^ Its prognosis is dismal, with a median survival of 8–21 months owing to its high recurrence, rapid progression, and widespread metastases.^1^ Additionally, due to its extreme rarity, there is no standard therapy. The current combination of surgery and aCT—adopted from well‐established therapeutic strategies for small cell lung cancer (SCLC)—is the most common treatments for patients with limited‐stage SCECs. This treatment was designed considering the histological and clinical similarity of SCECs and SCLCs.^2^ Despite initially high response rates, patients’ outcomes have been heterogeneous and many patients fail to benefit from this therapy while enduring its unnecessary side effects.^2^ Therefore, more precise and clinically feasible classification strategies are urgently needed to identify aCT responders.

To date, no studies have explored biomarkers for predicting the prognostic benefit after aCT in patients with SCECs. Antitumor immunity status is a critical determiner of chemotherapy and/or radiotherapy by our group and others.^3^, ^4^, ^5^ Given the importance of tumor‐infiltrating CD8^+^ T cells to antitumor immunity,^6^, ^7^ we evaluated the relationship between CD8^+^ T‐cell density and aCT benefit in patients with initial SCEC.

We retrospectively reviewed 196 patients initially diagnosed with SCEC who underwent surgery at the National Cancer Center (NCC) in China from 1984–2019. We accepted patients with mixed histology, provided more than 70% of the cells in the tumor section met SCEC criteria. After hematoxylin and eosin‐staining, cytokeratin detection (pancytokeratin AE1/AE3), and identification of neuroendocrine markers (synaptophysin and chromogranin A), we screened out 149 patients with available whole tumor sections and details about follow‐up information [relapse‐free survival (RFS) time and overall survival (OS) time]. Then 106 patients, treated with either surgery alone or surgery plus aCT, were filtered out for CD8+ T‐cell infiltration evaluation. The formalin‐fixed paraffin‐embedded whole tumor sections of these patients were assessed by immunohistochemical (IHC) staining of CD8 (clone C8/144B, Dako). Six cases were excluded by quality control before whole‐slide digital imaging. Finally, 100 cases—60 patients who received aCT and 40 patients who received only surgery—were enrolled in this study (Fig. S1). The clinicopathological features of these 100 patients are gathered in Table S1. We calculated the density of CD8+ T cells in the tumor regions using the HALO digital pathological platform. The representative IHC result of CD8 staining and the workflow of HALO analysis is displayed in Figure S2. All tests were two‐sided, and *P *< .05 indicated statistical significance.

The median number of CD8^+^ T‐cells for each patient with aCT treatment was 115.7 cells/mm^2^ (range 7.5–1297.3 cells/mm^2^) and the mean was 190.2 cells/mm^2^ (Table S1). Patients were subsequently divided into high (n = 20, ≥190.2/mm^2^) and low CD8 groups (n = 40, < 190.2/mm^2^). The relationships between CD8^+^ status and clinicopathologic characteristics for patients with aCT are summarized in Supplementary Table S2. High CD8 infiltration was significantly related to decreased cancer‐specific mortality (*P *= .003). A Kaplan‐Meier analysis with log‐rank test was introduced to investigate the impact of CD8 status on prognosis among those who received aCT. High CD8 infiltration was associated with prolonged OS (Figure 1A, HR = 0.2825, 95% CI 0.1379–0.5786, *P *= .0038) and RFS (Figure 1B, HR = 0.3231, 95% CI 0.1718–0.6076, *P *= .0016). To validate the prognostic performance of CD8 status, we plotted the time‐dependent receive operating characteristic (ROC) curve analysis for both OS and RFS. Results in Figure 1C showed that the CD8 status achieved the areas under curve (AUC) values of 0.704, 0.751, and 0.697 for predicting OS at 1‐, 3‐, and 5‐years. Meanwhile, the AUCs of CD8 status for predicting RFS at 1‐, 3‐, and 5‐years were 0.723, 0.698, and 0.687 (Figure 1D). We also compared the 3‐year ROC with tumor node metastasis (TNM) staging system. Results confirmed that the performance of CD8 status was superior to the TNM system for predicting both OS and RFS (Figure 1E,F). Then, to explore whether CD8 status was a significant independent predictor of prognosis for SCECs with aCT, univariate and multivariate Cox regression analyses were conducted. CD8 status was a significant predictor of both OS and RFS—independent of other covariables include sex, age, tumor location, macroscopic tumor type, tumor length, and TNM stage—in patients with SCEC who underwent aCT (Table S3).

**FIGURE 1:**
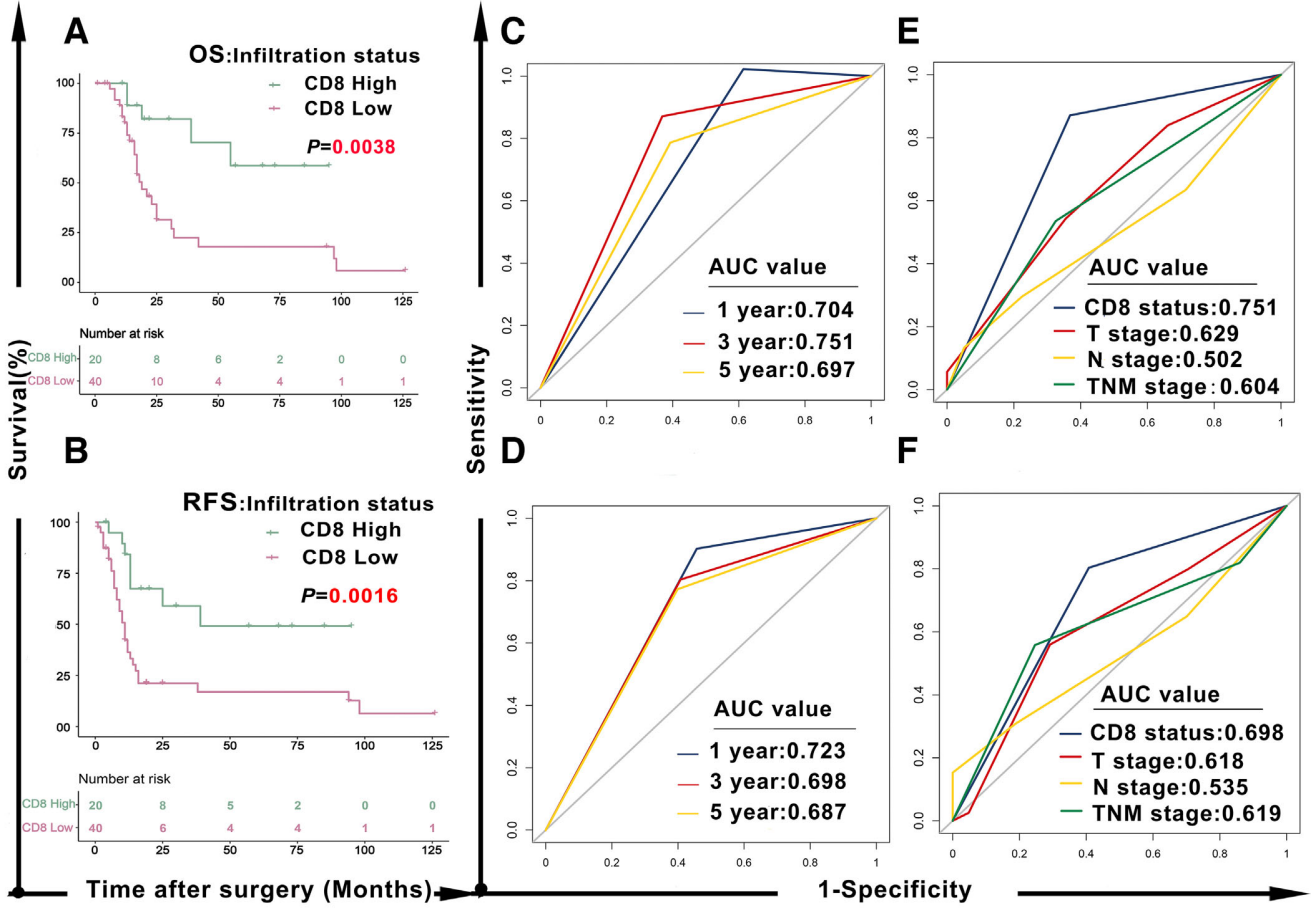
Tumor‐infiltrating CD8^+^ T cell status predicts aCT benefit in patients with SCEC. OS (A) and RFS (B) curves for patients with SCEC who underwent aCT according to CD8^+^ T cell infiltration status (High or Low); The 1‐, 3‐, and 5‐year ROC of the CD8 status for OS (C) and RFS (D) in patients with SCEC who underwent aCT; A comparison of 3‐year ROC curves for OS (E) and RFS (F) with TNM staging system showed the superiority of the CD8 status. aCT, adjuvant chemotherapy; SCEC, small cell esophageal carcinoma; OS, overall survival; RFS, relapse‐free survival

Given that the effects of surgery plus aCT in different SCEC populations compared with surgery alone, are controversial,^2^ we explored the survival rates of patients in different treatment groups. As shown in Figure 2A,B, there were no significant between‐group differences for OS and RFS (*P *> .05). Combining with the results mentioned above, we speculated that different CD8 statuses likely influence the survival benefits afforded by aCT. We further investigated whether only patients with high CD8 infiltration could acquire survival benefits to verify our hypothesis. Hence, the density of CD8 was also calculated in patients with surgery alone (n = 40) at the NCC (Table S1).

**FIGURE 2:**
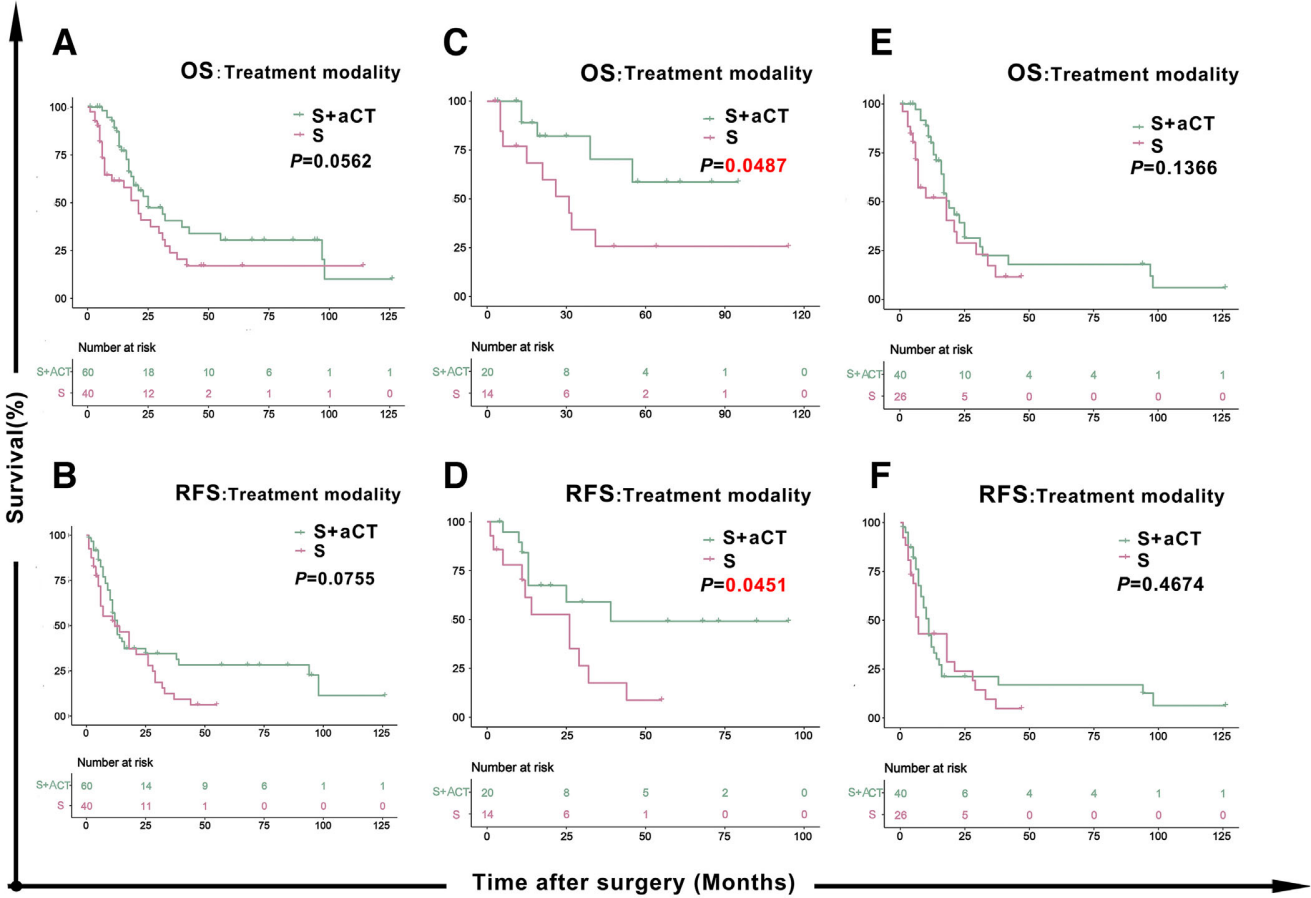
Tumor‐infiltrating CD8^+^ T cell status is a primary determinant for SCECs of benefiting from aCT. OS (A) and RFS (B) curves for patients with SCEC according to treatment modality (S or S+aCT); OS (C) and RFS (D) curves for patients with SCEC who had a high CD8 infiltration status according to treatment modality (S or S+aCT); OS (E) and RFS (F) curves for patients with SCEC who had a low CD8 infiltration status according to treatment modality (S or S+aCT). SCEC, small cell esophageal carcinoma; OS, overall survival; RFS, relapse‐free survival; S, surgery; aCT, adjuvant chemotherapy; ROC, receive operating characteristic; TNM, tumor node metastasis; HR, hazard ratio; CI, confidence interval

The median number of CD8^+^ T‐cells for all surgical patients was 121.0 cells/mm^2^ (range 11.1–1301.6 cells/mm^2^) and the mean was 191.1 cells/mm^2^. There was no significant difference in CD8^+^ T‐cell densities between surgery plus aCT and surgery groups (Figure S3). Using the same cut‐off value (190.2/mm^2^), for patients who underwent surgery, 14 patients were assigned to a high CD8 infiltration status subgroup and 26 patients were assigned to the low CD8 infiltration status subgroup. For the patients with high CD8 infiltration status, those who underwent aCT experienced significantly longer OS (Figure 2C, HR = 0.3538, 95% CI 0.1208–1.0360, *P *= .0487) and RFS (Figure 2D, HR = 0.4140, 95% CI 0.1625–1.0550, *P *= .0451) than those who received surgery alone. Among patients with low CD8 infiltration status, those who received aCT showed similar OS (Figure 2E) and RFS (Figure 2F) rates to those who underwent surgery alone (*P *> .05). These results suggest that patients with SCEC and high CD8 infiltration are more likely to benefit from aCT.

In conclusion, we identified the first biomarker—CD8 infiltration status—to predict the potential benefit and prognosis for SCECs with aCT. The absolute quantification of CD8^+^ T‐cells increases our findings' comparability and clinical applicability and may inform prognosis management for patients with SCECs. Patients with high CD8^+^ T‐cell infiltration should undergo systemic aCT. Patients with low CD8^+^ T‐cell infiltration should try other treatment schedules or perhaps join a clinical trial, rather than enduring the toxic side effects of, likely ineffective, aCT. CD8^+^ T‐cell infiltration status can be used to determine chemotherapy benefit and long‐term survival in patients with SCECs and may provide a rational basis for further investigation of the implementation of individualized combined immunotherapy and aCT. However, further prospective clinical trials are needed.

## CONFLICT OF INTEREST

The authors declare no conflict of interest.

## Supporting information

Supporting Information

Supporting Information

Supporting Information

Supporting Information
