# Supplementary material for: Tumor‐infiltrating CD8+ T cell is prognostic and predicts adjuvant chemotherapy benefit in patients with limited‐stage small cell esophageal carcinoma
Source: Clin Transl Med. 2021 Jun 27;11(6):e456. doi: 10.1002/ctm2.456 (PMC8236121; doi:10.1002/ctm2.456)

**Supplementary Figure 1.** Flow chart of the study;


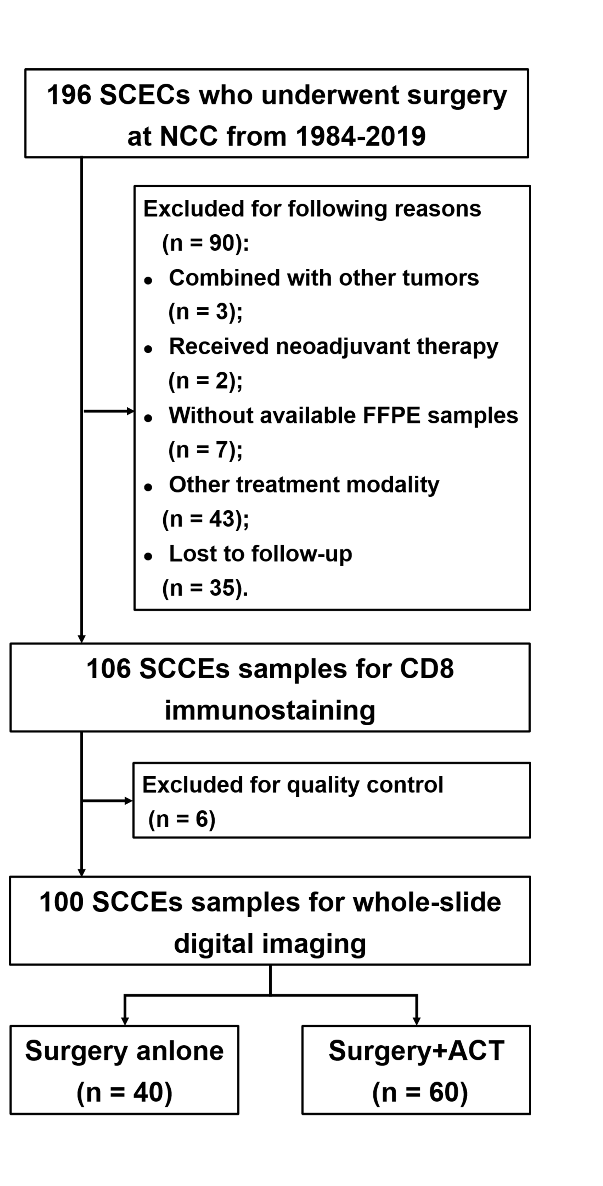


**Supplementary Figure 2.** CD8+ T-cell infiltration and the workflow of HALO analysis. (a), the representative IHC result of CD8 staining, left panel (×10), right panel (×20); (b), the workflow of HALO system to detect and count of CD8+ T cells and to the area of tumor region. IHC, immunohistochemical.


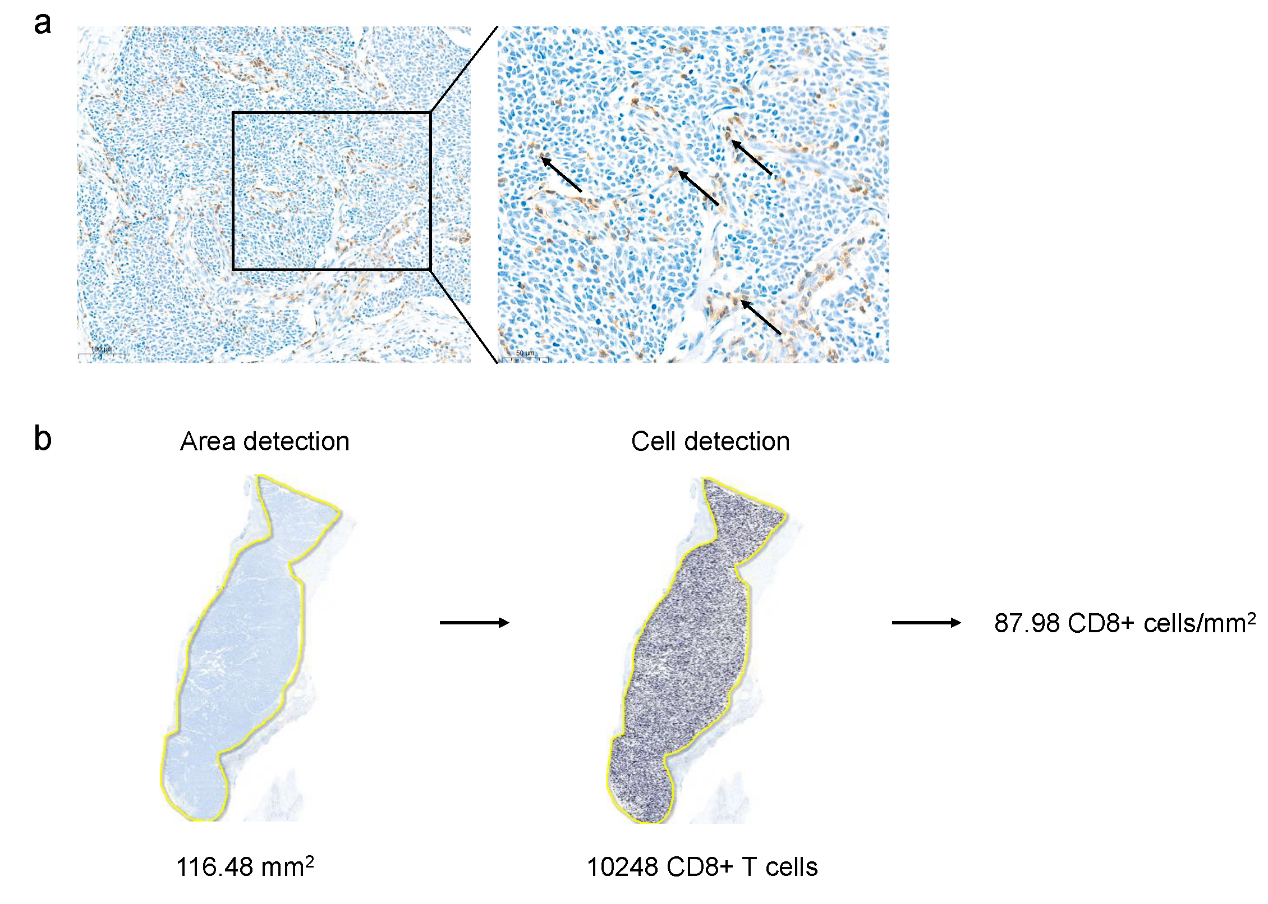


**Supplementary Figure 3.** The distributions of CD8+ T-cell density from different groups in patients with small cell esophageal carcinoma. S, surgery; ACT, adjuvant chemotherapy.


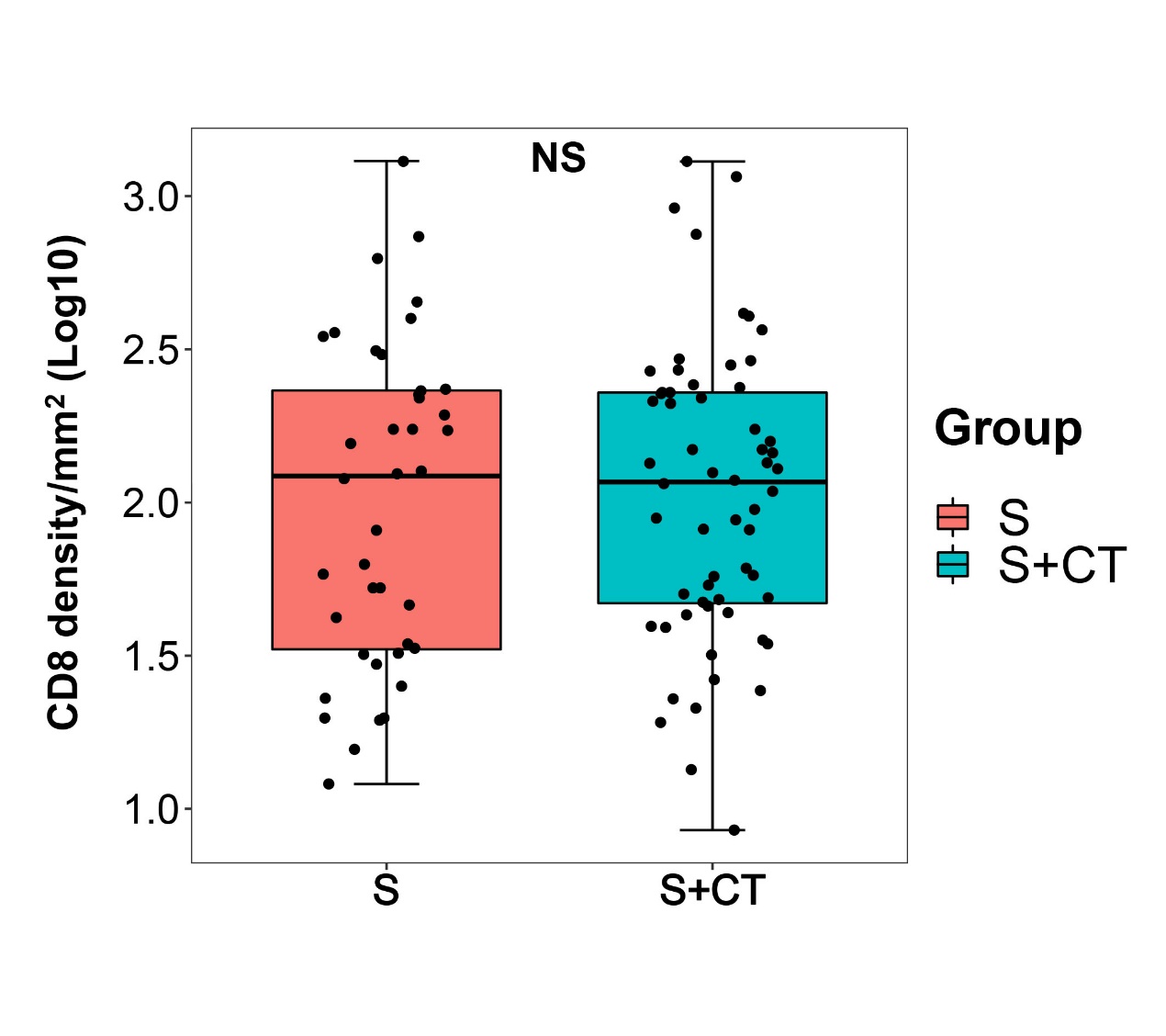

Supplement: Supplementary file 1 — Supporting Information [file CTM2-11-e456-s002.docx]
