# Supplementary material for: Tumor‐infiltrating CD8+ T cell is prognostic and predicts adjuvant chemotherapy benefit in patients with limited‐stage small cell esophageal carcinoma
Source: Clin Transl Med. 2021 Jun 27;11(6):e456. doi: 10.1002/ctm2.456 (PMC8236121; doi:10.1002/ctm2.456)
Supplement: Supplementary file 2 — Supporting Information [file CTM2-11-e456-s003.docx]

**Supplementary Table 1**. Cohort characteristics of 100 patients with limited-stage small cell esophageal carcinoma.

| Characteristics | Surgery+ACT  (*N* = 60) |  |  | Surgery alone  (*N* = 40) |  |
| --- | --- | --- | --- | --- | --- |
|  | No. of patients | % |  | No. of patients | % |
| Sex |  |  |  |  |  |
| Male | 46 | 76.7% |  | 26 | 65.0% |
| Female | 14 | 23.3% |  | 14 | 35.0% |
| Age, y |  |  |  |  |  |
| ≤60 | 30 | 50.0% |  | 23 | 57.5% |
| >60 | 30 | 50.0% |  | 17 | 42.5% |
| Location |  |  |  |  |  |
| Upper | 5 | 8.3% |  | 4 | 10.0% |
| Middle | 47 | 78.3% |  | 32 | 80.0% |
| Lower | 8 | 13.3% |  | 4 | 10.0% |
| Length, cm |  |  |  |  |  |
| <5 | 37 | 61.7% |  | 24 | 60.0% |
| ≥5 | 23 | 38.3% |  | 16 | 40.0% |
| Macroscopic tumor type |  |  |  |  |  |
| Superficial, Protruded | 14 | 23.3% |  | 6 | 15.0% |
| Medullary, Mushroom, Ulcerative, Intracavity | 46 | 76.7% |  | 34 | 85.0% |
| T stage |  |  |  |  |  |
| T1 | 14 | 23.3% |  | 14 | 35.0% |
| T2 | 18 | 30.0% |  | 13 | 32.5% |
| T3 | 26 | 43.3% |  | 13 | 32.5% |
| T4 | 2 | 3.3% |  | 0 | 0.0% |
| N stage |  |  |  |  |  |
| N0 | 20 | 33.3% |  | 17 | 42.5% |
| N1 | 24 | 40.0% |  | 13 | 32.5% |
| N2 | 10 | 16.7% |  | 8 | 20.0% |
| N3 | 6 | 10.0% |  | 2 | 5.0% |
| TNM stage |  |  |  |  |  |
| I | 10 | 16.7% |  | 10 | 25.0% |
| II | 23 | 38.3% |  | 13 | 32.5% |
| III | 27 | 45.0% |  | 17 | 42.5% |
| CD8 density/mm^2^ |  |  |  |  |  |
| Median | 115.7 |  |  | 121.0 |  |
| Range | 7.5-1,297.3 |  |  | 11.1–1,301.6 |  |

Abbreviations: ACT, adjuvant chemotherapy.
