# Supplementary material for: Tumor‐infiltrating CD8+ T cell is prognostic and predicts adjuvant chemotherapy benefit in patients with limited‐stage small cell esophageal carcinoma
Source: Clin Transl Med. 2021 Jun 27;11(6):e456. doi: 10.1002/ctm2.456 (PMC8236121; doi:10.1002/ctm2.456)
Supplement: Supplementary file 3 — Supporting Information [file CTM2-11-e456-s001.docx]

**Supplementary Table 2**. Infiltration of CD8+ T-cells and clinicopathological features in surgery plus adjuvant chemotherapy cohort.

| Clinicopathological features | Total  (*N* = 60) |  | CD8 density/mm^2^ | | |
| --- | --- | --- | --- | --- | --- |
|  |  |  | <190.2 (Low) | >190.2 (High) | *P* value |
|  |  |  | (*N* =40, 66.6%) | (*N* = 20, 33.3%) |  |
| Sex, *n* (%) |  |  |  |  |  |
| Male | 46 (76.7) |  | 31 (51.7) | 15 (25.0) |  |
| Female | 14 (23.3) |  | 10 (16.7) | 4 (6.7) |  |
| Age, y, *n* (%) |  |  |  |  |  |
| ≤60 | 30 (50.0) |  | 18 (30.0) | 12 (20.0) |  |
| >60 | 30 (50.0) |  | 22 (36.7) | 8 (13.3) |  |
| Location, *n* (%) |  |  |  |  |  |
| Upper | 5 (8.3) |  | 4 (6.7) | 1 (1.7) |  |
| Middle | 47 (78.3) |  | 31 (51.7) | 16 (26.7) |  |
| Lower | 8 (13.3) |  | 5 (8.3) | 3 (5.0) |  |
| Length, cm, *n* (%) |  |  |  |  |  |
| <5 | 37 (61.7) |  | 23 (38.3) | 14 (23.3) |  |
| ≥5 | 23 (38.3) |  | 17 (28.3) | 6 (10.0) |  |
| Macroscopic tumor type, *n* (%) |  |  |  |  |  |
| Superficial/Protruding | 14 (23.3) |  | 8 (13.3) | 6 (10.0) |  |
| Medullary/Mushroom/Ulcerative/Intracavity | 46 (76.7) |  | 32 (53.3) | 14 (23.3) |  |
| T stage, *n* (%) |  |  |  |  |  |
| T1 | 14 (23.3) |  | 6 (10.0) | 8 (13.3) |  |
| T2/T3/T4 | 46 (76.7) |  | 34 (56.7) | 12 (20.0) |  |
| N stage, *n* (%) |  |  |  |  |  |
| N0 | 20 (33.3) |  | 15 (25.0) | 5 (8.3) |  |
| N1/N2/N3 | 40 (66.7) |  | 25 (49.7) | 15 (5.0) |  |
| TNM stage, *n* (%) |  |  |  |  |  |
| I | 10 (16.7) |  | 6 (10.0) | 4 (6.7) |  |
| II | 23 (38.3) |  | 13 (21.7) | 10 (16.7) |  |
| III | 27 (45.0) |  | 21 (35.0) | 6 (10.0) |  |
| Cancer-specific mortality, *n* (%) |  |  |  |  | 0.003 |
| Yes | 31 (51.7) |  | 26 (43.3) | 5 (8.3) |  |
| No | 29 (48.3) |  | 14 (23.3) | 15 (25.0) |  |
