# Supplementary material for: Tumor‐infiltrating CD8+ T cell is prognostic and predicts adjuvant chemotherapy benefit in patients with limited‐stage small cell esophageal carcinoma
Source: Clin Transl Med. 2021 Jun 27;11(6):e456. doi: 10.1002/ctm2.456 (PMC8236121; doi:10.1002/ctm2.456)
Supplement: Supplementary file 4 — Supporting Information [file CTM2-11-e456-s004.docx]

**Supplementary Table 3**. Univariable and multivariate Cox regression of CD8 status and clinicopathological characteristics and survival in SCCEs who underwent ACT

| Variable | OS | | | | |  | RFS | | | | |
| --- | --- | --- | --- | --- | --- | --- | --- | --- | --- | --- | --- |
|  | Univariate | |  | Multivariate | |  | Univariate | |  | Multivariate | |
|  | HR  (95% CI) | *P* value |  | HR  (95% CI) | *P* value |  | HR  (95% CI) | *P* value |  | HR  (95% CI) | *P* value |
| Sex  Male/Female | 0.857  (0.325-2.261) | 0.755 |  | 0.498  (0.156-1.593） | 0.240 |  | 0.975  (0.426-2.230) | 0.952 |  | 0.531  (0.193-1.467) | 0.222 |
| Age  >60/≤60 | 1.348  (0.657-2.768) | 0.416 |  | 0.984  (0.443-2.187） | 0.969 |  | 1.677  (0.877-3.208) | 0.118 |  | 1.362  (0.665-2.788) | 0.398 |
| Macroscopic tumor type  Medullary, Mushroom, Ulcerative, Intracavity/Superficial, Protruding | 1.461  (0.626-3.408) | 0.380 |  | 0.843  (0.227-3.123) | 0.798 |  | 1.880  (0.825-4.281) | 0.133 |  | 1.661  (0.456-6.047) | 0.441 |
| Tumor location  Middle, Upper/Lower | 0.492  (0.206-1.174) | 0.110 |  | 0.376  (0.135-1.047） | 0.061 |  | 0.657  (0.313-1.378) | 0.266 |  | 0.811  (0.313-2.103) | 0.667 |
| Tumor length (cm)  ≥5/<5 | 1.208  (0.591-2.467) | 0.604 |  | 0.876  (0.338-2.273) | 0.785 |  | 1.450  (0.766-2.744) | 0.253 |  | 1.224  (0.542-2.768) | 0.627 |
| T stage  T2, T3, T4/T1 | 1.602  (0.651-3.944) | 0.305 |  | 0.396  (0.071-2.207） | 0.290 |  | 1.756  (0.769-4.008) | 0.181 |  | 0.350  (0.085-1.432) | 0.144 |
| N stage  N1, N2, N3/N0 | 0.940  (0.448-1.976) | 0.871 |  | 0.250  (0.049-1.268) | 0.094 |  | 1.323  (0.666-2.625) | 0.424 |  | 1.085  (0.262-4.491) | 0.910 |
| TNM. Stage  II, III/I | 1.532  (0.891-2.633) | 0.123 |  | 3.557  (0.769-16.444) | 0.104 |  | 1.765  (1.066-2.922) | 0.027 |  | 1.609  (0.415-6.232) | 0.491 |
| CD8+ T cell infiltration status  High/Low | 0.264  (0.100-0.697) | 0.007 |  | 0.268  (0.080-0.898) | 0.033 |  | 0.304  (0.138-0.669) | 0.003 |  | 0.277  (0.096-0.794) | 0.017 |

Abbreviations: OS, overall survival; RFS, relapse free survival; SCEC, small cell esophageal carcinoma; HR, hazard ratio; CI, confidence interval.
